# Supplementary material for: Dendritic autophagy degrades postsynaptic proteins and is required for long-term synaptic depression in mice
Source: Nat Commun. 2022 Feb 3;13:680. doi: 10.1038/s41467-022-28301-z (PMC8814153; doi:10.1038/s41467-022-28301-z)

**Supplementary Information**

# Dendritic autophagy degrades postsynaptic proteins and is required for long-term synaptic depression in mice

Emmanouela Kallergi[1#], Akrivi-Dimitra Daskalaki[1#], Angeliki Kolaxi[1], Come Camus[2], Evangelia Ioannou[3], Valentina Mercaldo[1], Per Haberkant[4], Frank Stein[4], Kyriaki Sidiropoulou[3], Yannis Dalezios[5,6], Mikhail M Savitski[4,7], Claudia Bagni[1,8], Daniel Choquet[2,9], Eric Hosy[2] and Vassiliki Nikoletopoulou[1*]

[1]Department of Fundamental Neurosciences, University of Lausanne, Lausanne, 1005, Switzerland; [2]University of Bordeaux, CNRS, Interdisciplinary Institute for Neuroscience, IINS, UMR 5297, F-33000 Bordeaux, France; [3]School of Biological Sciences, University of Crete, Heraklion, 70013, Greece; [4]Proteomic Core Facility (PCF), European Molecular Biology Laboratory (EMBL), Heidelberg, Germany; [5]School of Medicine, University of Crete, Heraklion, 71003, Greece; [6]Institute of Applied and Computational Mathematics (IACM), Foundation for Research and Technology - Hellas (FORTH), Heraklion, Greece. [7]Genome Biology Unit, European Molecular Biology Laboratory (EMBL); [8]Department of Biomedicine and Prevention, University of Rome Tor Vergata, 00133, Rome, Italy; [9]University of Bordeaux, CNRS, INSERM, Bordeaux Imaging Center, BIC, UMS 3420, US 4, F-33000 Bordeaux, France.

# equal contribution

*Corresponding author: Vassiliki Nikoletopoulou (vassiliki.nikoletopoulou@unil.ch)

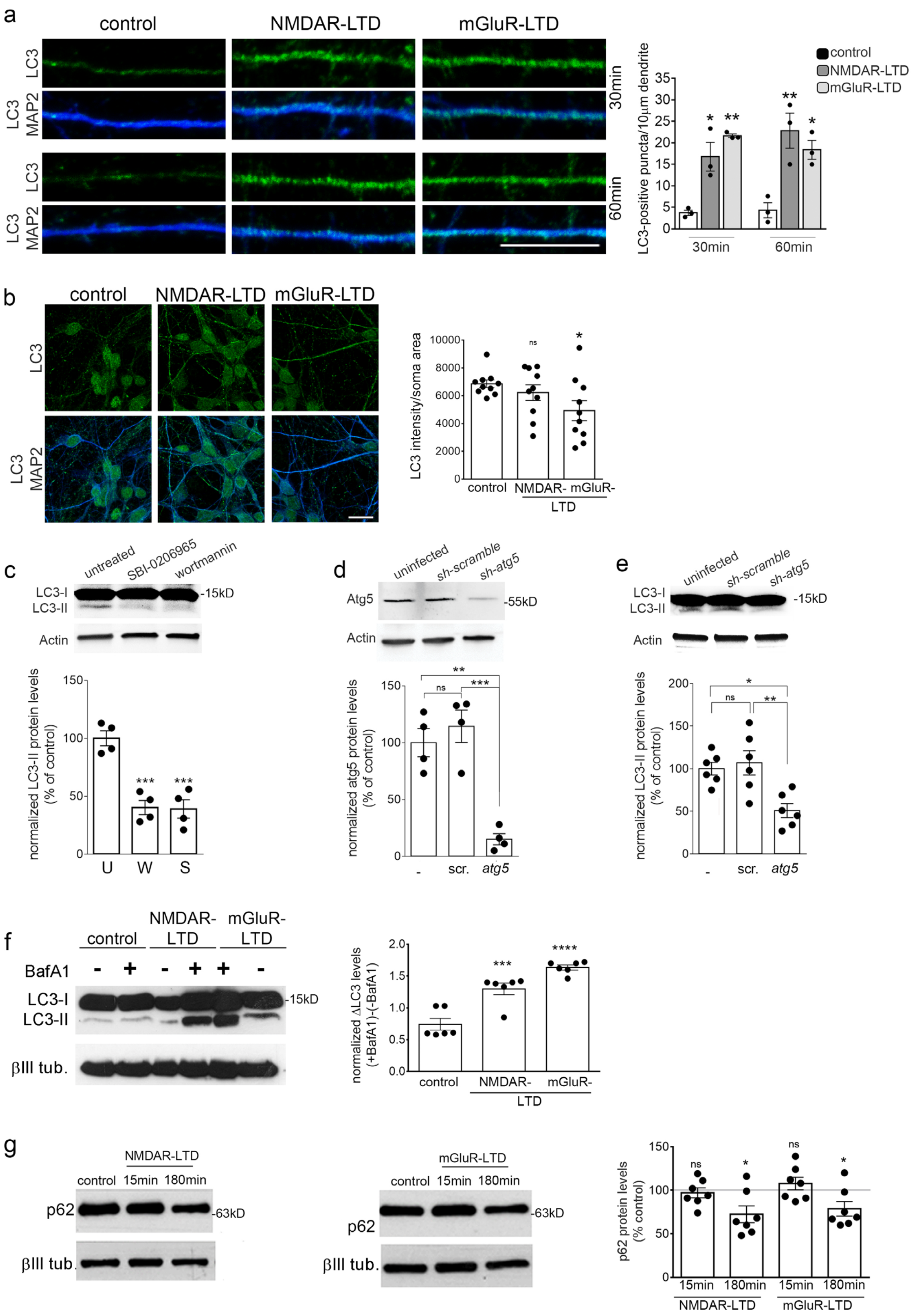
a
control
NMDAR-LTD
mGluR-LTD
LC3
LC3 MAP2
30min
60min
LC3-positive puncta/10μm dendrite
control
NMDAR-LTD
mGluR-LTD
30min
60min
b
control
NMDAR-LTD
mGluR-LTD
LC3
LC3 MAP2
LC3 intensity/soma area
ns
control
NMDAR-
mGluR-
LTD
c
untreated
SBI-0206965
wortmannin
LC3-I
LC3-II
-15kD
Actin
normalized LC3-II protein levels (% of control)
U
W
S
d
uninfected
sh-scramble
sh-atg5
Atg5
-55kD
Actin
normalized atg5 protein levels (% of control)
ns
scr.
atg5
e
uninfected
sh-scramble
sh-atg5
LC3-I
LC3-II
-15kD
Actin
normalized LC3-II protein levels (% of control)
ns
scr.
atg5
f
control
NMDAR-LTD
mGluR-LTD
BafA1
LC3-I
LC3-II
-15kD
βIII tub.
normalized ΔLC3 levels (+BafA1)-(-BafA1)
control
NMDAR-
mGluR-
LTD
g
NMDAR-LTD
control 15min 180min
p62
-63kD
βIII tub.
mGluR-LTD
control 15min 180min
p62
-63kD
βIII tub.
p62 protein levels (% control)
ns
15min
180min
NMDAR-LTD
mGluR-LTD

**Figure S1. Chemical NMDAR- and mGluR-LTD trigger the rapid appearance of autophagic structures in dendrites of cultured neurons.**

**a,** Representative confocal images of dendrites of cultured neurons stained with antibodies against LC3 and MAP2 under control conditions or 30 and 60 minutes after NMDAR-LTD and mGluR-LTD. Graph showing the number of LC3-positive puncta per 10μm dendrite length for each time-point. Bars represent mean values +/- SEM. N=3 independent experiments per treatment. Statistical analyses were performed by one-way ANOVA. (F(5,12)=11.91, P=0.0003) (Tukey's multiple comparison test $P_{control/30\text{-}NMDA/30}$=0.0257 $P_{control/30\text{-}DHPG/30}$=0.0025, $P_{NMDA/30\text{-}DHPG/30}$=0.7277, $P_{control/60\text{-}NMDA/60}$=0.0019, $P_{control/60\text{-}DHPG/60}$=0.0158, $P_{NMDA/60\text{-}DHPG/60}$=0.7925) Scale bar: 10μm.

**b,** Representative confocal images of cultured neurons stained with antibodies against LC3 and MAP2 under control conditions or 15 minutes after NMDAR-LTD and mGluR-LTD. Graph showing the LC3 intensity normalized to soma area for each condition. Bars represent mean values +/- SEM. N=10 independent experiments per treatment. Statistical analyses were performed by one-way ANOVA. (F(2,27)=3.221, P=0.0556) (Tukey's multiple comparison test $P_{control\text{-}NMDA}$=0.6987, $P_{control\text{-}DHPG}$=0.0490, $P_{NMDA\text{-}DHPG}$=0.2325) Scale bar: 10μm.

**c,** Western blot analysis for LC3 and actin in lysates from untreated control neurons or after an one-hour treatment with wortmannin (500nM) or SBI-0206569 (500nM). Graph showing the normalized LC3-II levels in the different treatments. Bars represent mean values +/- SEM. N=4 independent experiments per treatment. Statistical analyses were performed by one-way ANOVA. (F(2,9)=25,58, P=0.0002) (Tukey's multiple comparison test $P_{U\_W}$=0.0005, $P_{U\_S}$=0.0004, $P_{W\_S}$=0.9910).

**d,** Western blot analysis for Atg5 and actin in lysates from uninfected neurons, and neurons infected with *sh-scramble* or *sh-atg5* constructs. Graph showing the normalized Atg5 levels in the different conditions. Bars represent mean values +/- SEM. N=4 independent experiments per treatment. Statistical analyses were performed by one-way ANOVA. (F(2,9)=22,68,

P=0,0003) (Tukey's multiple comparison test $P_{control\_scr}$=0,6514, $P_{control\_shatg5}$=0.0012, $P_{scr\_shatg5}$ =0.0004).

**e,** Western blot analysis for LC3 and actin in lysates from uninfected neurons, and neurons infected with *sh-scramble* or *sh-atg5* constructs. Graph showing the normalized LC3-II levels in the different conditions. Bars represent mean values +/- SEM. N=6 independent experiments per treatment. Statistical analyses were performed by one-way ANOVA. (F(2,15)=8,618, P=0,0032) (Tukey's multiple comparison test $P_{control\_scr}$=0.8888, $P_{control\_shatg5}$=0.0117, $P_{scr\_shatg5}$ =0.0046).

**f,** Western blot analysis for LC3 and βIII-tubulin in lysates prepared from neurons subjected to NMDAR- or mGluR-LTD and in the presence or absence of 50nM BafilomycinA1 during the pulse and for 1 hour after. Graph showing the normalized ΔLC3-II levels (+BafA1) - (-BafA1) under control and chemical LTD conditions. Bars represent mean values +/- SEM. N=6 independent experiments per condition. Statistical analyses were performed by one-way ANOVA, F(2,15)=33.68, P<0.0001 (Tukey's multiple comparison test $P_{control\_NMDA}$=0.0004, $P_{control\_DHPG}$<0.0001).

**g,** Western blot analysis of p62 and βIII-tubulin in lysates prepared from neurons subjected to either control or chemical induction of NMDAR- or mGluR- LTD, 15 and 180 minutes after the pulses. Graph showing the p62 protein levels after NMDAR- and mGluR-LTD normalized to percentage of the control. Bars represent mean values +/- SEM. N=7 independent experiments per condition. Statistical analyses were performed by Student's *t*-test. For NMDAR-LTD 15min P=0.5477, for NMDAR-LTD 180min P=0.0175, mGluR-LTD 15min P=0.4698, for mGluR-LTD 180min P=0.0332.

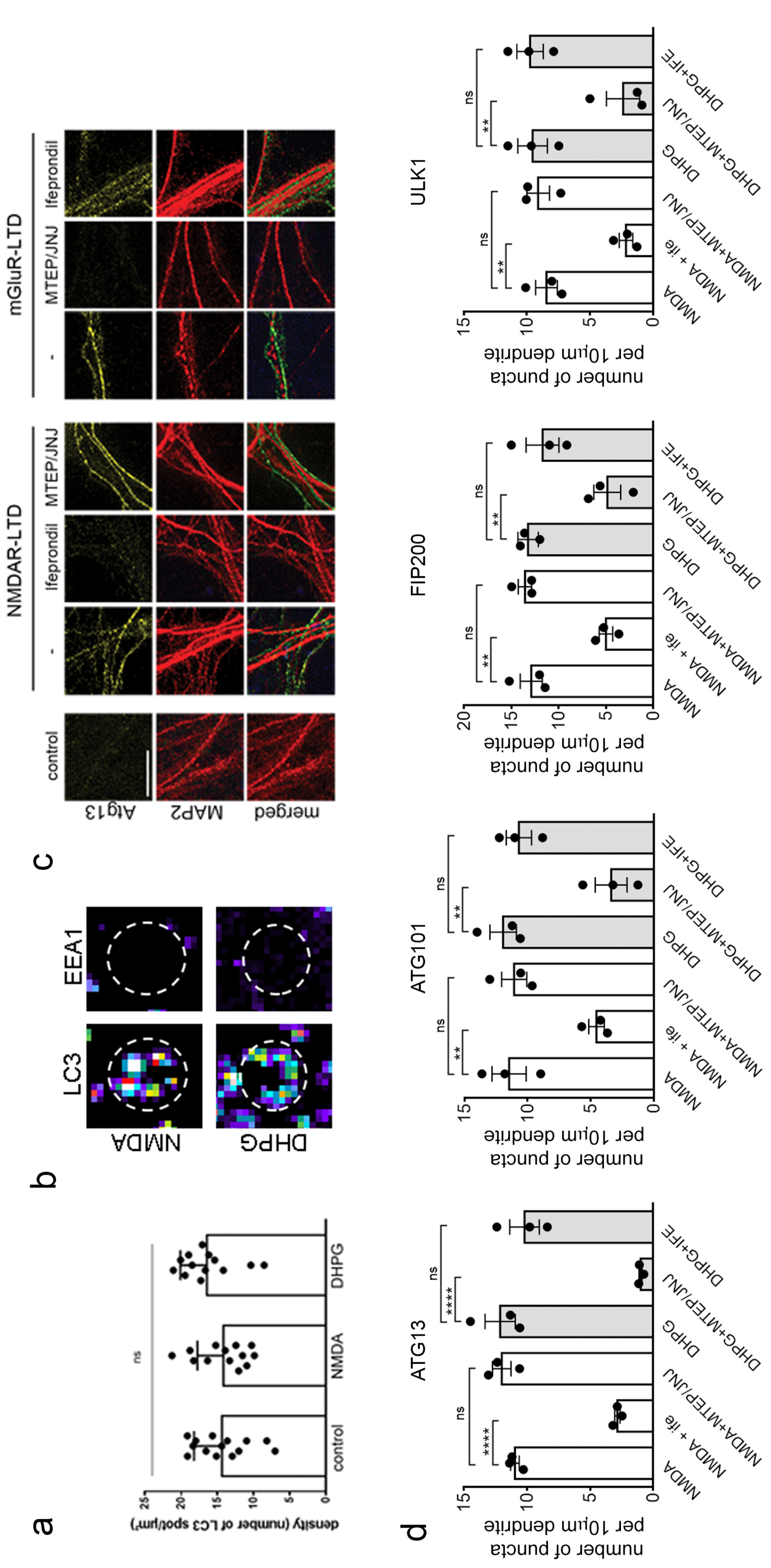
a
density (number of LC3 spot/μm²)
control
NMDA
DHPG
ns
b
NMDA
DHPG
LC3
EEA1
c
NMDAR-LTD
mGluR-LTD
control
Ifeprondil
MTEP/JNJ
Atg13
MAP2
merged
d
ATG13
ATG101
FIP200
ULK1
number of puncta per 10μm dendrite
NMDA
NMDA + ife
NMDA+MTEP/JNJ
DHPG
DHPG+MTEP/JNJ
DHPG+IFE
ns
**
****

**Figure S2. Autophagic vesicles are locally generated in dendrites following LTD.**

**a,** Graph indicating the density (the number of spots per $\mu m^2$) of LC3 either in control or upon chemical LTD conditions, which was not associated with a phagophore structure. Bars represent mean values +/- SEM. n=13-14 dendrites per condition, from N=3 independent experiments. Statistical analyses were performed by one-way ANOVA, $F(2,33)=0.3572$, $P=0.7$.

**b, R**epresentative super resolution microscopy dSTORM image of a secondary dendrite labeled with an antibody against LC3 and the early endosome marker EEA1, 15 minutes after cLTD. (N=3 independent experiments with similar results).

**c,** Representative confocal images of cultured neurons immunolabeled with antibodies against Atg13 and MAP2 (dendrites), in control or LTD conditions and in the presence or absence of Ifenprodil (10μM) or MTEP (10μM)/JNJ16259685 (10μM) for 1 hour before, during and 15 minutes after the pulses. Scale bar: 20μm. (N=3 independent experiments with similar results).

**d,** Graphs indicating the number of Atg13, Atg101, ULK1 and FIP200 puncta per 10μm of dendrite under the aforementioned conditions. Bars represent mean values +/- SEM. N=3 independent experiments per condition. Statistical analyses were performed using one-way ANOVA. Atg13: $F(5,12)=41.55$, $P<0.0001$. Tukey's multiple comparison test, $P_{NMDA\_NMDA/IFE}<0.0001$, $P_{NMDA\_NMDA/MTEP-JNJ}=0.9216$, $P_{DHPG\_DHPG/IFE}=0.5082$, $P_{DHPG\_DHPG/MTEP-JNJ}<0.0001$. Atg101: $F(5,12)=12.69$, $P=0.0002$. Tukey's test, $P_{NMDA\_NMDA/IFE}=0.0066$, $P_{NMDA\_NMDA/MTEP-JNJ}=0.9998$, $P_{DHPG\_DHPG/IFE}=0.9580$, $P_{DHPG\_DHPG/MTEP-JNJ}=0.0012$. FIP200: $F(5,12)=13.11$, $P=0.0002$. Tukey's test, $P_{NMDA\_NMDA/IFE}=0.0039$, $P_{NMDA\_NMDA/MTEP-JNJ}=0.9981$, $P_{DHPG\_DHPG/IFE}=0.9277$, $P_{DHPG\_DHPG/MTEP-JNJ}=0.0024$. ULK1: $F(5,12)=13.03$, $P=0.0002$. Tukey's test, $P_{NMDA\_NMDA/IFE}=0.0080$, $P_{NMDA\_NMDA/MTEP-JNJ}=0.9966$, $P_{DHPG\_DHPG/IFE}>0.9999$, $P_{DHPG\_DHPG/MTEP-JNJ}=0.0029$.

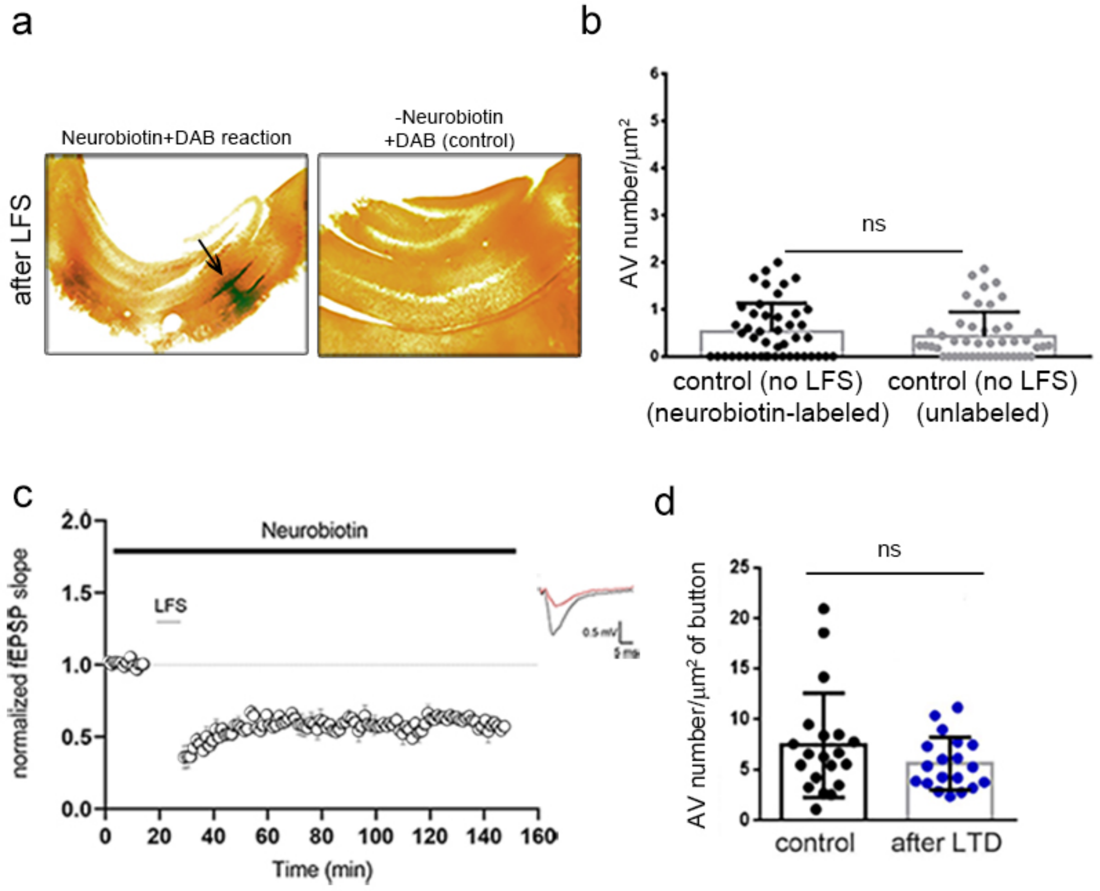


**Figure S3. LTD triggers dendritic autophagy in hippocampal slices.**

**a,** Representative images of 70μm thick hippocampal slices with or without neurobiotin labeling after LFS protocol, visualized using the ABC – DAB reaction method to show the specificity of the labeling in the recorded CA1 area. (N=3 independent experiments with similar results).

**b,** Graph indicating the number of autophagosomes per μm$^2$ in control conditions (without LFS induction) between neurobiotin labeled and unlabeled adjacent dendrites. Bars represent mean values +/- SEM. n=46 dendrites per condition (N=3 animals). Statistical analysis was performed using unpaired, two-tailed Student's *t*-test, P=0.5706.

**c,** Time plot of the normalized fEPSP slope in wild-type P22-P28 hippocampal slices following LFS, using a recording electrode filled with 1.5% neurobiotin in 2M NaCl. N=3 animals. Dots represent mean values +/- SEM.

**d,** Graph indicating the number of autophagosomes per µm2 of buttons that synapse onto neurobiotin-labelled dendrites in control conditions and after LFS-induced LTD. Bars represent mean values +/- SEM. N=3 animals per condition, and 5-7 buttons per animal. Statistical analysis was performed using unpaired, two-tailed Student's t-test, P=0.1792.

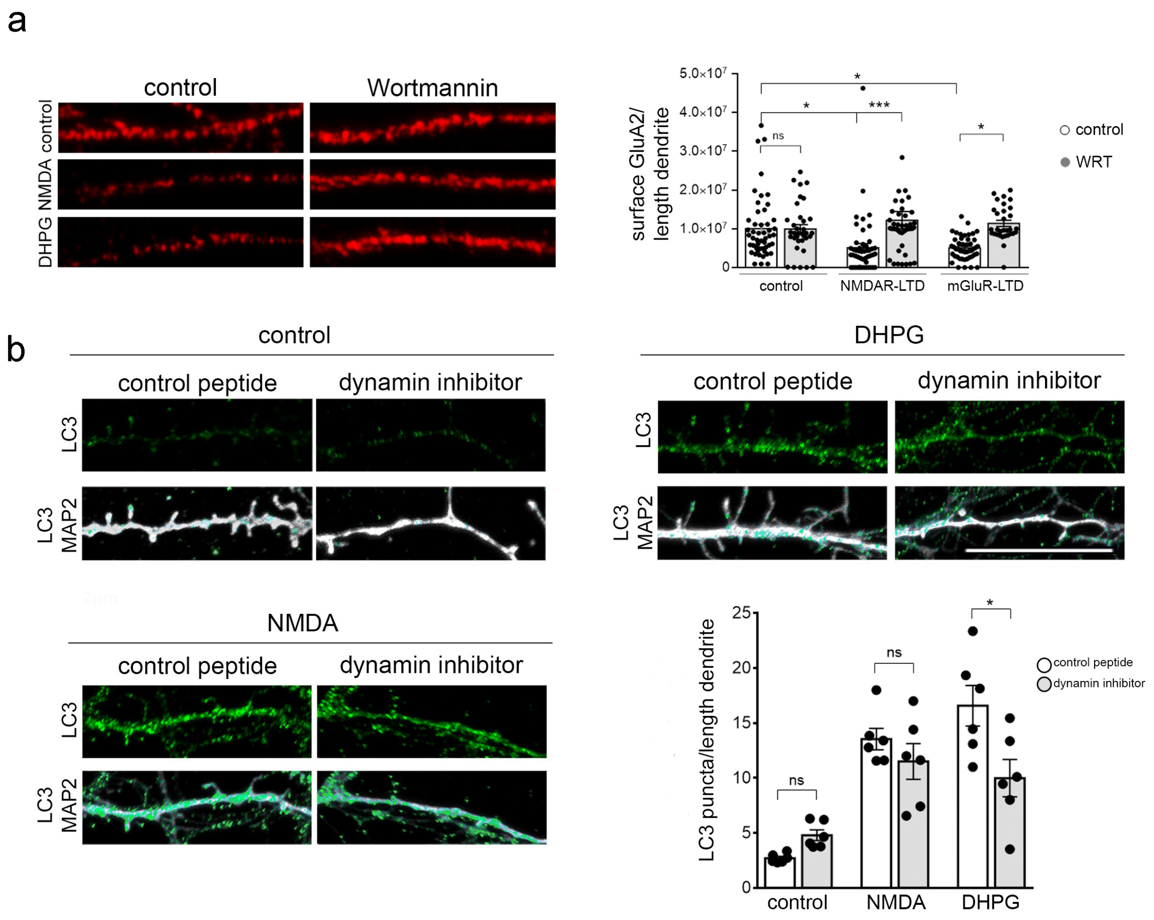


**Figure S4. Autophagy degrades AMPARs and its scaffold during LTD.**

**a,** Confocal images of dendrites immuno-labeled with an antibody against the extracellular region of GluA2 under control conditions or 15 minutes after LTD induction and in the presence or not of wortmannin (500nM). Wortmannin was applied 25 minutes before, during and 15 minutes after the pulses. Scale bar: 10μm. Graph showing the surface labeling of GluA2, normalized to dendritic length. Bars represent mean values +/- SEM. N=6 independent experiments (n=5-7 dendrites per experiment, 30-40 dendrites in total). Statistical analysis was performed using one-way ANOVA ($F(5, 248) = 6.073$, $P<0.0001$) (Tukey's test $P_{control-NMDA}=0.0452$, $P_{control-DHPG}=0.0450$, $P_{control-control/WRT}>0.99$, $P_{NMDA-NMDA/WRT}=0.001$, $P_{DHPG-DHPG/WRT}=0.0167$).

**b**, Representative confocal images of cultured neurons immunolabeled with antibodies against LC3 and MAP2, either before or 15 minutes after chemical induction of NMDAR- and mGluR-LTD, and in the presence of a dynamin inhibitory peptide or a control peptide. Scale bar:

10μm. Graph showing the number of LC3-positive puncta per length of dendrite. Bars represent mean values +/- SEM. N=6 independent experiments per condition. Statistical analyses were performed using two-way ANOVA. $F(5,25)=14.39$, $P<0.0001$. Tukey's multiple comparison test, $P_{NMDA\_NMDA/DYN}= 0.9010$, $P_{DHPG\_DHPG/DYN}=0.0269$, $P_{control\_control/DYN}=0.8906$.

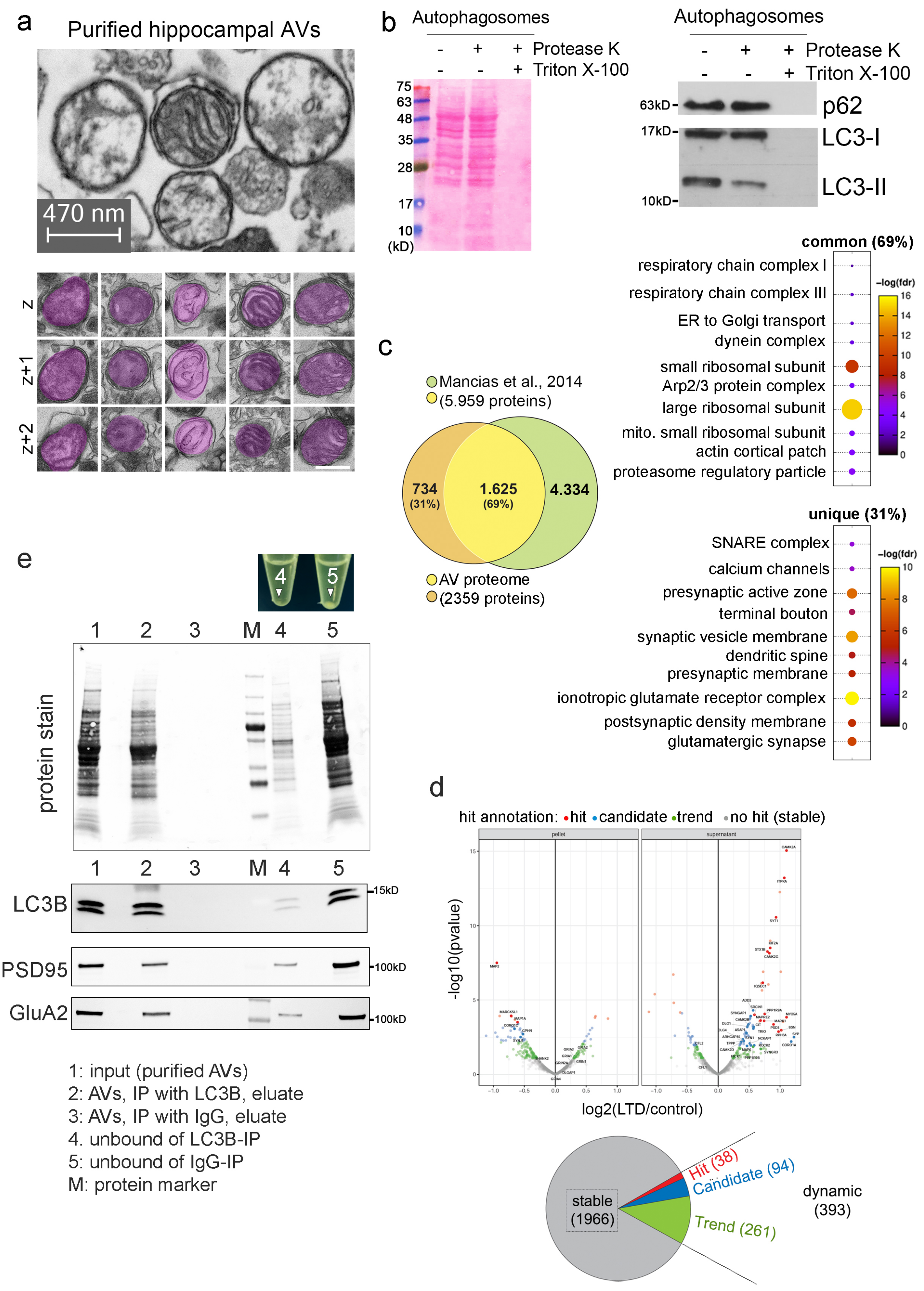
a
Purified hippocampal AVs
470 nm
z
z+1
z+2
b
Autophagosomes
- + + Protease K
- - + Triton X-100
75
63
48
35
28
17
10
(kD)
Autophagosomes
- + + Protease K
- - + Triton X-100
63kD
p62
17kD
LC3-I
LC3-II
10kD
c
Mancias et al., 2014
(5.959 proteins)
734 (31%)
1.625 (69%)
4.334
AV proteome
(2359 proteins)
common (69%)
respiratory chain complex I
respiratory chain complex III
ER to Golgi transport
dynein complex
small ribosomal subunit
Arp2/3 protein complex
large ribosomal subunit
mito. small ribosomal subunit
actin cortical patch
proteasome regulatory particle
−log(fdr)
unique (31%)
SNARE complex
calcium channels
presynaptic active zone
terminal bouton
synaptic vesicle membrane
dendritic spine
presynaptic membrane
ionotropic glutamate receptor complex
postsynaptic density membrane
glutamatergic synapse
−log(fdr)
d
hit annotation: • hit • candidate • trend • no hit (stable)
pellet
supernatant
-log10(pvalue)
log2(LTD/control)
stable (1966)
Hit (38)
Candidate (94)
Trend (261)
dynamic (393)
e
1 2 3 M 4 5
protein stain
LC3B
15kD
PSD95
100kD
GluA2
100kD
1: input (purified AVs)
2: AVs, IP with LC3B, eluate
3: AVs, IP with IgG, eluate
4. unbound of LC3B-IP
5: unbound of IgG-IP
M: protein marker

**Figure S5. Proteomic profiling of the autophagic cargo during LTD.**

**a,** Representative electron micrographs of the purified AV preparation, showing that it is comprised of intact, double membrane-bound vesicles. Scale bar top panel: 470nm, bottom panel: 250nm. (N=3 independent experiments with similar results).

**b,** Ponceau staining of Western blot membrane containing purified autophagic vesicles that are untreated or treated with Proteinase K and Triton X-100, as indicated. Western blot analysis of purified autophagic vesicles (untreated or treated with Proteinase K and Triton X-100), with antibodies against LC3 and p62. Note that p62, a known cargo protein, is fully protected from Proteinase K digestion, indicating that vesicles are intact. Also note that LC3-II levels are reduced by half after proteinase K treatment, consistent with its localization both on the outer (PK-sensitive) and inner (PK-protected) membranes of autophagic vesicles.

**c,** Pie chart showing the proportion of the hippocampal slice autophagic cargo that is common with the autophagic cargo previously described in cultured cell lines in the Mancias et al., 2014 study. Graphs showing the cell component analysis, as false discovery rate (FDR)-corrected p-values, of the common cargo (69% of hippocampal slice cargo) and of the uncommon or unique cargo (31% of hippocampal slice cargo), as compared to the Mancias et al., 2014 study.

**d,** Volcano plot analysis of the proteins found in pellet and supernatant fractions of purified AVs. Proteins depicted in grey consist the stable autophagic cargo between control and NMDAR-LTD AVs, while in red, blue and green the dynamic. Red represents hits with a value of as false discovery rate (FDR)-corrected p-values <0.05, blue represents candidates with a value of fdr<0.2 and green represents trends with a value of fdr<0.6. Pie chart showing the proportion of the autophagic cargo that is stable between baseline and NMDAR-LTD conditions (grey), or dynamic in LTD (red, blue and green). Color code is as in (c). Numbers indicate the actual number of proteins in each category.

**e,** Protein stain and Western blot analysis with antibodies for LC3B, PSD95 and GluA2 of a membrane with the indicated samples, which represent the input (lane 1, purified AV fraction), the eluates after an immunoprecipitation with LC3B antibody or IgG control (lanes 2 and 3,

respectively) and the corresponding unbound materials (lanes 4 and 5). Picture insert of Eppendorf tubes indicates the material that is pelleted after centrifugation of the unbound materials and loaded in lanes 4 and 5.

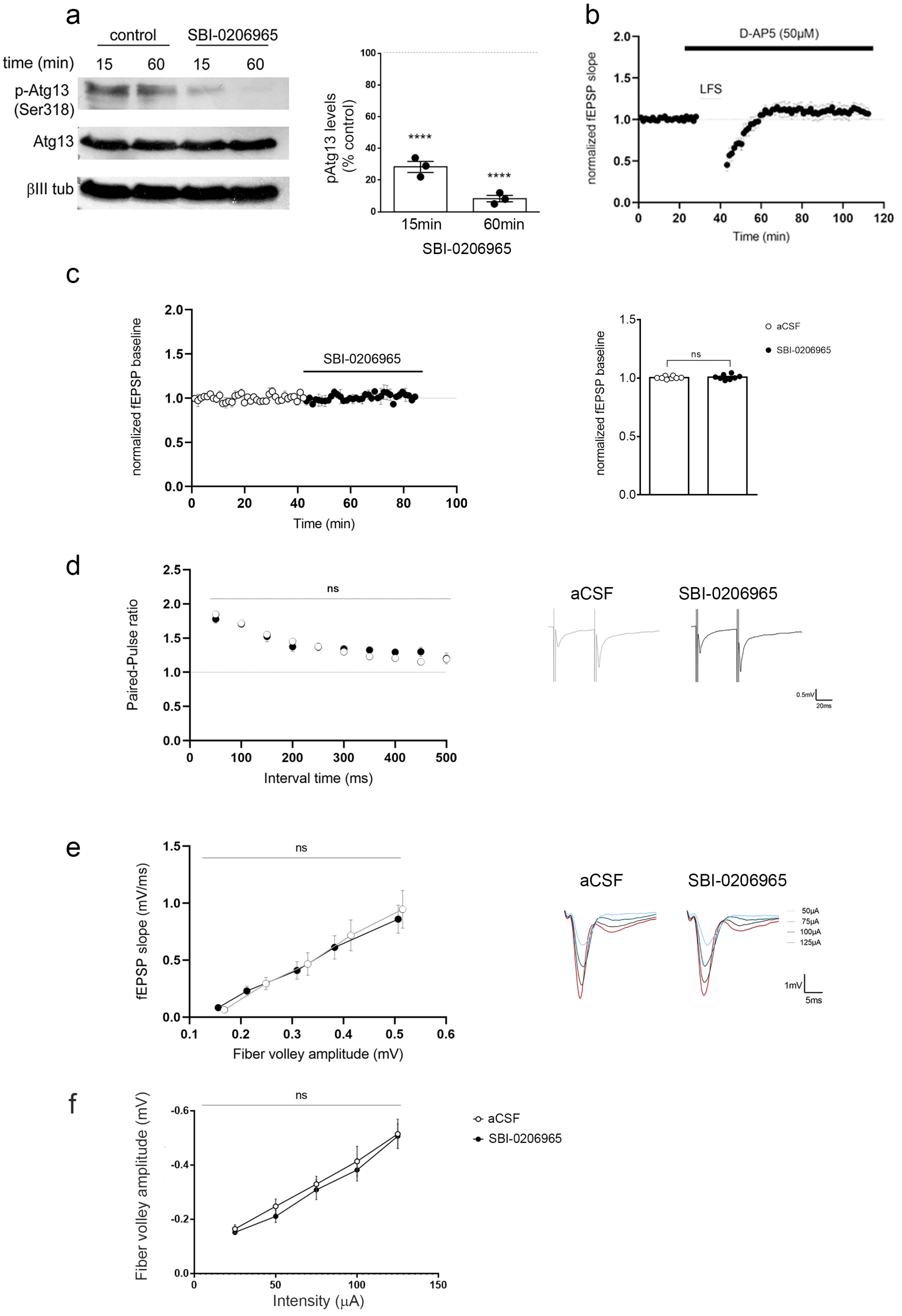
a
control
SBI-0206965
time (min)
15
60
15
60
p-Atg13
(Ser318)
Atg13
βIII tub
pAtg13 levels
(% control)
****
****
15min
60min
SBI-0206965
b
D-AP5 (50µM)
LFS
normalized fEPSP slope
Time (min)
c
SBI-0206965
normalized fEPSP baseline
Time (min)
aCSF
SBI-0206965
ns
d
ns
Paired-Pulse ratio
Interval time (ms)
aCSF
SBI-0206965
0.5mV
20ms
e
ns
fEPSP slope (mV/ms)
Fiber volley amplitude (mV)
aCSF
SBI-0206965
50µA
75µA
100µA
125µA
1mV
5ms
f
ns
Fiber volley amplitude (mV)
Intensity (µA)
aCSF
SBI-0206965

**Figure S6. Inhibition of autophagic vesicle biogenesis prevents NMDAR- and mGluR-LTD.**

**a,** Western blot analysis of pSer318-Atg13, total Atg13 and β-III tubulin from lysates of wild-type P22 hippocampal slices treated, by bath application, with vehicle or with SBI-0206965 (500nM) for 15 and 60 minutes. Graph showing the normalized to the percentage of control protein levels of pSer318-Atg13 after treatment with SBI-0206965 in the indicated time points. Bars represent mean values +/- SEM. N=3 independent experiments per condition. Statistical analysis was performed using unpaired, two-tailed Student's *t*-test (for 15min P=0.0001 and for 60min, P<0.0001).

**b,** Time-plot of normalized fEPSP slope before and after LTD induction by LFS protocol (1200 pulses at 1.4Hz) in the presence of NMDA-R antagonist D-AP5 (50μM). N=4 animals.

**c,** Time-plot of normalized fEPSP baseline and bar graphs of the average normalized fEPSP baseline first under control conditions (aCSF; white bar) and subsequently during bath application of SBI-0206965 (1μM) (black bar). Baseline fEPSPs for both conditions are normalized to control baseline. Bars represent mean values +/- SEM. N=8 animals per condition. Statistical analysis was performed using unpaired, two-tailed Student's *t*-test, comparing the average normalized baseline responses of the SBI-0206965 treated slices to the control (P=0.6258).

**d,** Graph showing the paired-pulse ratio at different inter-stimulus intervals from control (aCSF; white circles) or bath application of SBI-0206965 (black circles). Bars represent mean values +/- SEM. N=8 animals per condition. Statistical analyses were performed using two-way repeated measures ANOVA (F(1,14)=0.6889, P=0.4205).

**e,** Graph showing the fEPSP slope and fiber volley amplitude relationship under control conditions (white circles) or bath application of SBI-0206965 (black circles). N=8 animals per condition. Statistical analysis was performed using two-way repeated-measures ANOVA (F (1,70)=0.9685, P=0.3284). Representative fEPSP traces evoked by increasing stimulation intensities under control conditions (aCSF) or bath application of SBI-0206965.

f, Graph showing the fiber volley amplitude and intensity relationship under control conditions (white circles) or bath application of SBI-0206965 (black circles). N=8 animals per condition. Statistical analysis was performed using two-way repeated-measures ANOVA ($F_{(1,70)}=0.9272$, $P=0.3389$).

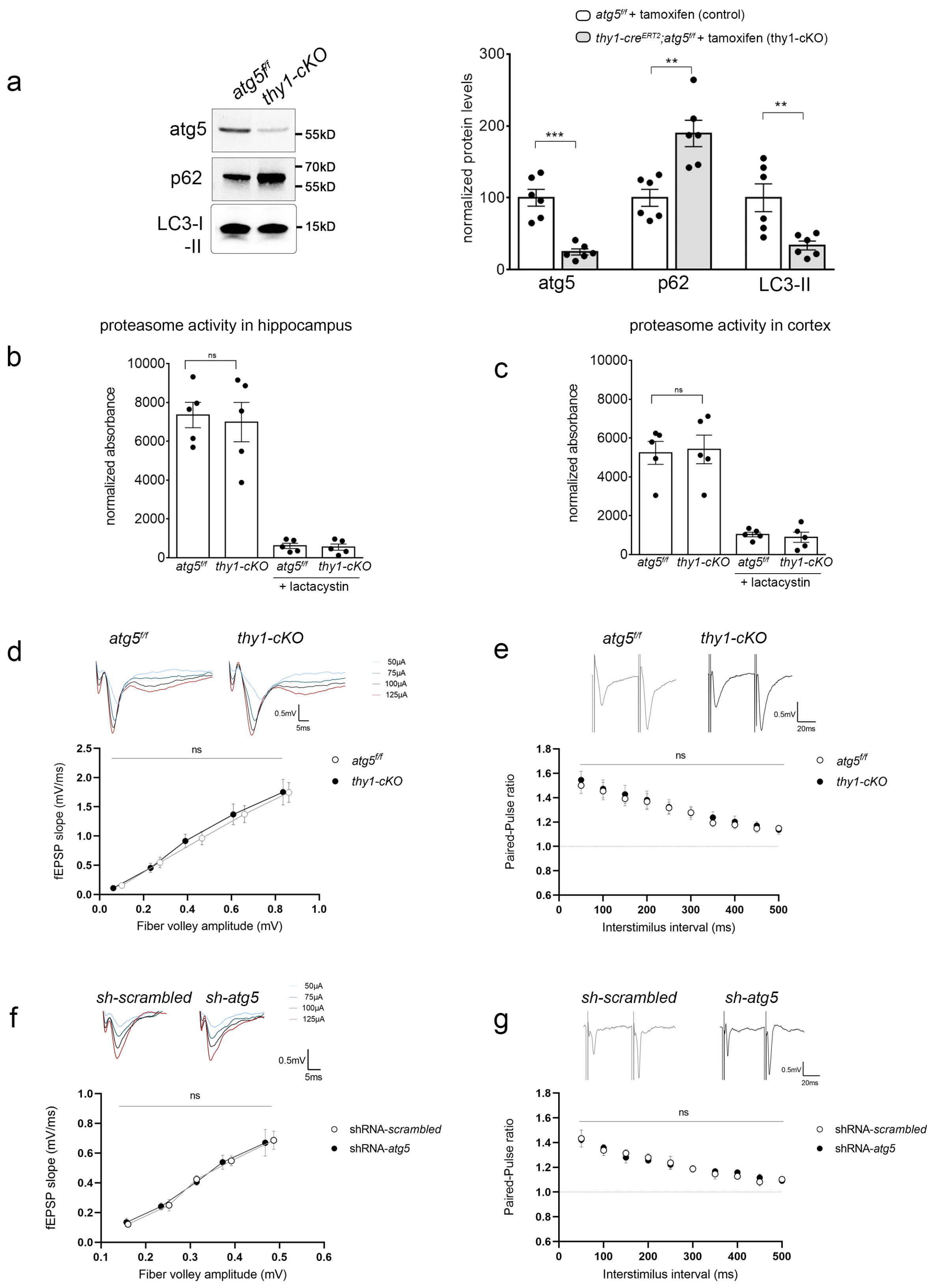
a
atg5f/f
thy1-cKO
atg5
p62
LC3-I
-II
55kD
70kD
55kD
15kD
atg5f/f + tamoxifen (control)
thy1-creERT2;atg5f/f + tamoxifen (thy1-cKO)
normalized protein levels
atg5
p62
LC3-II
b
proteasome activity in hippocampus
normalized absorbance
atg5f/f
thy1-cKO
+ lactacystin
c
proteasome activity in cortex
d
0.5mV
5ms
50μA
75μA
100μA
125μA
fEPSP slope (mV/ms)
Fiber volley amplitude (mV)
e
Paired-Pulse ratio
Interstimulus interval (ms)
20ms
f
sh-scrambled
sh-atg5
shRNA-scrambled
shRNA-atg5
g
ns

**Figure S7. Autophagy is required cell-autonomously in postsynaptic pyramidal neurons for LTD.**

**a,** Western blot analysis in P22 hippocampal lysates from tamoxifen-treated *atg*$^{f/f}$ mice (control), and *thy1-creERT2;atg*$^{f/f}$ *(thy1-cKO)*, with an antibody against Atg5, p62 and LC3. Graph showing the Atg5, p62 and LC3-II protein levels in the two genotypes, normalized to total protein levels. Bars represent mean values +/- SEM. N=6 animals per genotype. Statistical analyses were performed by unpaired, two-tailed Student's *t*-test (atg5, P=0.0001; p62, P=0.0022; LC3B-II, P=0.0085).

**b,** Graph showing the proteasomal activity, expressed as normalized absorbance, of hippocampal lysates from P40 control and *thy1-cKO* mice. Lactacystin was added as a control, and it efficiently blocked the activity of the proteasome in all samples. Bars represent mean values +/- SEM. N=5 animals per genotype. Statistical analyses were performed by unpaired, two-tailed Student's *t*-test (P=0.7692). **c,** Same as in (b) for cortical lysates (P=0.8557).

**d**, fEPSP slope and fiber volley amplitude relationship in P22 hippocampal slices of tamoxifen-treated control (white circles) or *thy1-cKO* (black circles) animals. N=9 animals per genotype. Statistical analysis was performed using two-way repeated-measures ANOVA (F (1,80) = 0.08050, P=0.7774). Representative fEPSP traces evoked by increasing stimulation intensities in control and *thy1-cKO* animals.

**e,** Graphs showing the paired-pulse ratio at different inter-stimulus intervals in P22 hippocampal slices from tamoxifen-treated control (white circles) and *thy1-cKO* (black circles) animals. Bars represent mean values +/- SEM. N=6 animals per genotype. Statistical analyses were performed using two-way repeated-measures ANOVA, comparing the paired pulse ratio of the *thy1-cKO* to the control, at the different interstimulus intervals. F(1,10)=0.09837, P=0.7602.

**f,** fEPSP slope and fiber volley amplitude relationship in slices expressing *AAV-pCamk2-shscrambled-mCherry* (white circles) and *AAV-pCamk2-shatg5-eGFP* (black circles) in the CA1 area. N=6 animals per condition. Statistical analysis was performed using two-way repeated-measures ANOVA (F (1,50) =0.07601, P=0.7839). Representative fEPSP traces

evoked by increasing stimulation intensities obtained from slices from the two aforementioned conditions.

**g,** Graph showing the paired-pulse ratio at different inter-stimulus intervals in adult slices expressing *AAV-pCamk2-shscrambled-mCherry* (white circles) and *AAV-pCamk2-shatg5-eGFP* (black circles) in the CA1 area. Bars represent mean values +/- SEM. N=6 animals per condition. Statistical analyses were performed using two-way repeated-measures ANOVA, comparing the paired pulse ratio of the sh-*atg5* to sh-*scrambled* controls, at the different inter-stimulus intervals. $F(1,10)=0.0001230$, $P=0.9914$.

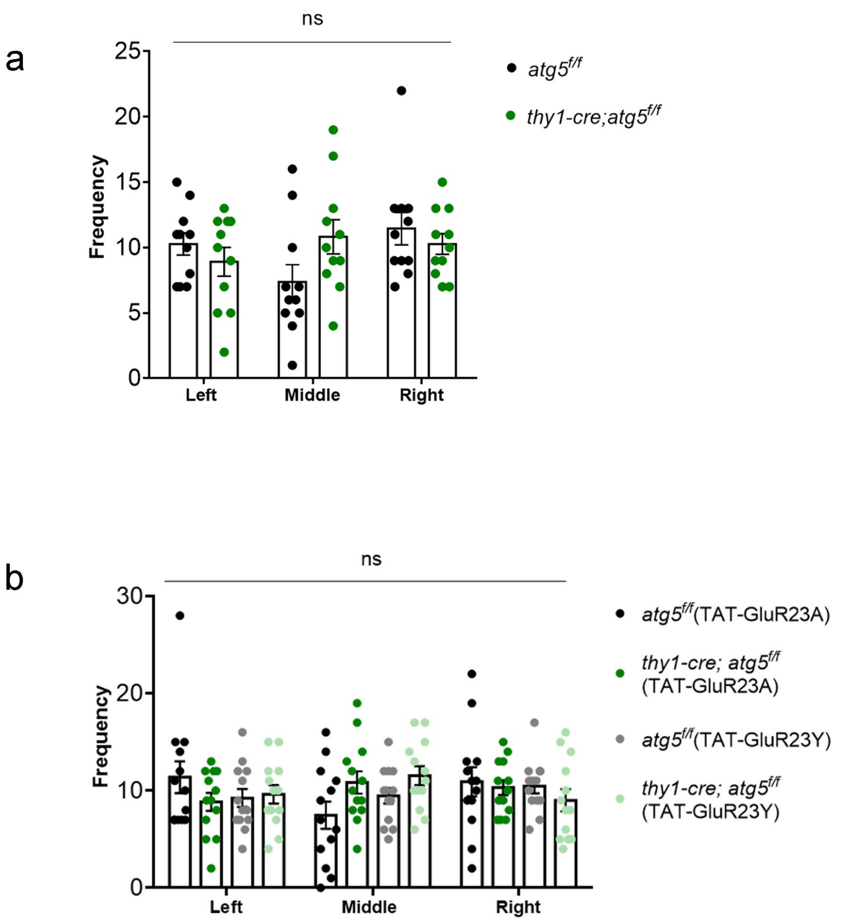


**Figure S8. Autophagy deficiency in excitatory neurons alters cognitive flexibility.**

**a,** Graph showing the number of entries from the left, middle or right entrance of the cognition wall during the first thirty entries, after the beginning of the protocol between *atg5$^{f/f}$* and *thy1;atg5$^{f/f}$* animals (N=11 animals per genotype). Bars represent mean values of +/-SEM. Statistical analysis was performed by two-way ANOVA (F (1,60) =0.1098, P=0.7416).

**b,** Same as a, but among *atg5$^{f/f}$* and *thy1;atg5$^{f/f}$* with administration of TAT-GluR23Y or TAT-GluR23A, as a control peptide. These measurements demonstrate the preference, if any, of mice for a specific entry before the beginning of the learning phase. Bars represent mean values of +/-SEM. (N=13 animals per genotype). Statistical analysis was performed by two-way ANOVA (F (3,144)= 0,05189, P=0.9843).

## Source data files for Supplementary Figures

### Figure S1

**Figure S1c**

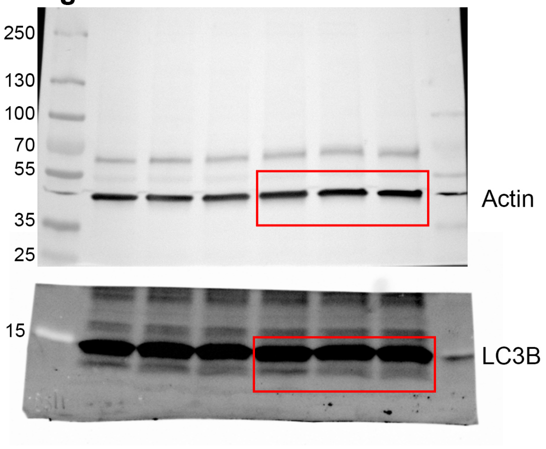


**Figure S1d**

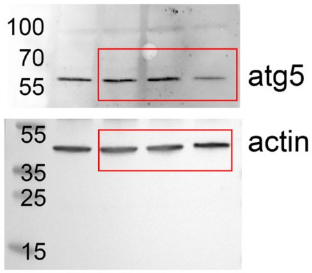


**Figure S1e**

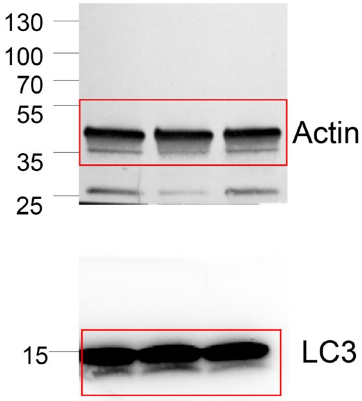


**Figure S1f**

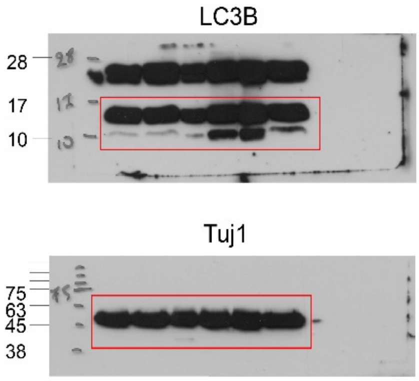


**Figure S1g**

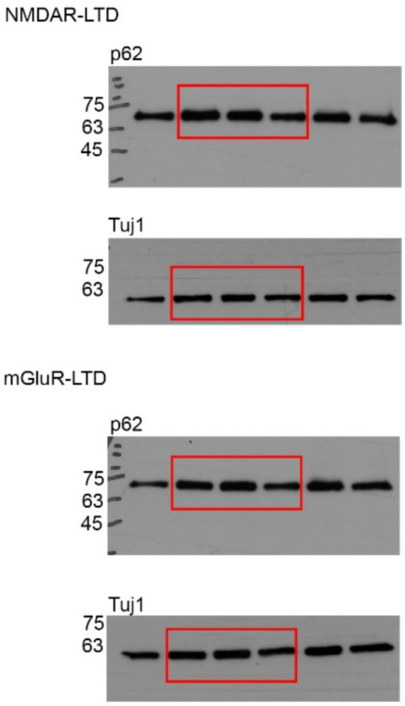

Figure S5

## Figure S5b

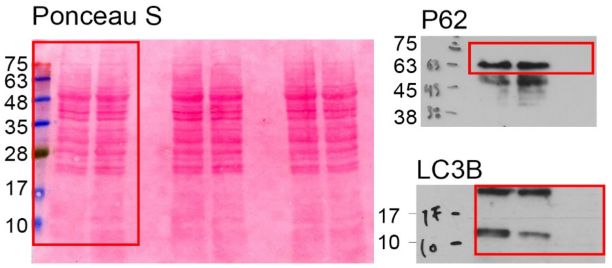


## Figure S5e

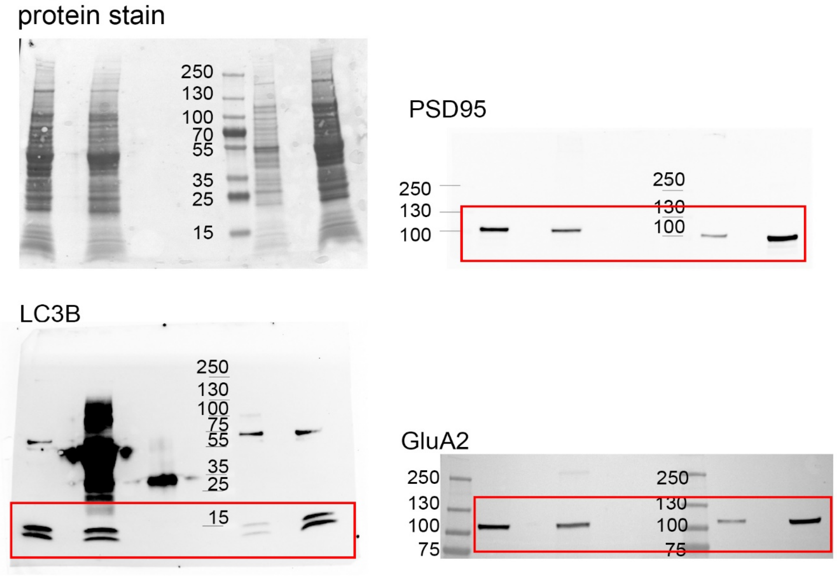

Figure S6

**Figure S6a**

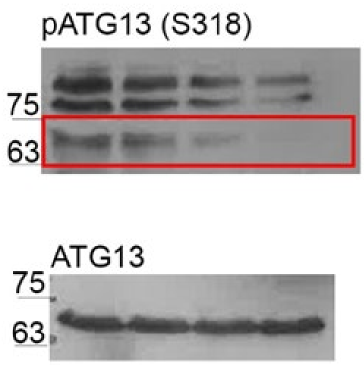


Figure S7

**Figure S7a**

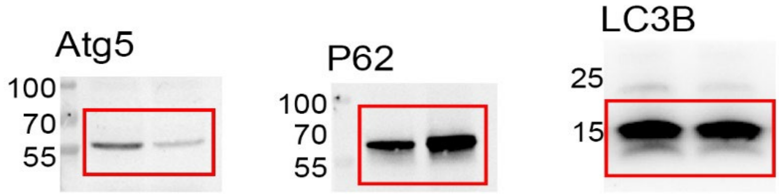

Supplement: Supplementary file 1 — Supplementary Information file [file 41467_2022_28301_MOESM1_ESM.pdf]
