# Supplementary material for: Dendritic autophagy degrades postsynaptic proteins and is required for long-term synaptic depression in mice
Source: Nat Commun. 2022 Feb 3;13:680. doi: 10.1038/s41467-022-28301-z (PMC8814153; doi:10.1038/s41467-022-28301-z)
Supplement: Supplementary file 2 — Reporting Summary [file 41467_2022_28301_MOESM2_ESM.pdf]

nature portfolio

Corresponding author(s): Vassiliki Nikoletopoulou

Last updated by author(s): 05 December 2021

Reporting Summary

Nature Portfolio wishes to improve the reproducibility of the work that we publish. This form provides structure for consistency and transparency in reporting. For further information on Nature Portfolio policies, see our Editorial Policies and the Editorial Policy Checklist.

Statistics

For all statistical analyses, confirm that the following items are present in the figure legend, table legend, main text, or Methods section.

n/a	Confirmed	
☐	☒	The exact sample size (*n*) for each experimental group/condition, given as a discrete number and unit of measurement
☐	☒	A statement on whether measurements were taken from distinct samples or whether the same sample was measured repeatedly
☐	☒	The statistical test(s) used AND whether they are one- or two-sided<br>*Only common tests should be described solely by name; describe more complex techniques in the Methods section.*
☒	☐	A description of all covariates tested
☒	☐	A description of any assumptions or corrections, such as tests of normality and adjustment for multiple comparisons
☐	☒	A full description of the statistical parameters including central tendency (e.g. means) or other basic estimates (e.g. regression coefficient) AND variation (e.g. standard deviation) or associated estimates of uncertainty (e.g. confidence intervals)
☐	☒	For null hypothesis testing, the test statistic (e.g. F, t, r) with confidence intervals, effect sizes, degrees of freedom and P value noted<br>*Give P values as exact values whenever suitable.*
☒	☐	For Bayesian analysis, information on the choice of priors and Markov chain Monte Carlo settings
☒	☐	For hierarchical and complex designs, identification of the appropriate level for tests and full reporting of outcomes
☒	☐	Estimates of effect sizes (e.g. Cohen's d, Pearson's r), indicating how they were calculated

Our web collection on statistics for biologists contains articles on many of the points above.

Software and code

Policy information about availability of computer code

Data collection	All confocal images were aquired using the Zeiss LSM900 (Zen blue 2019 software), Leica TCS SP8 (LAS AF 3 software) and Nikon Ti2 | Yokogawa CSU-W1 (Nikon NIS-Elements AR 5.0 software). Electrophysiological recordings were acquired with pClamp 10 SoftwareSuite (Molecular Devises LLC). Electron microscopy images were acquired with JEM-2100 transmission electron microscope (JEOL Ltd, Akishima, Tokyo, JAPAN). Mass spectrometry data were acquired by LC-MS/MS on a Q Exactive Plus (Thermo Scientific). STORM data were acquired with LEICA DMi8 microscope. Video tracking for behavioral experiments was conducted by EthoVision XT.
Data analysis	Image J 1.53C, GraphPad Prism 8, Adobe Photoshop, pClamp 10 SoftwareSuite (Molecular Devises LLC), IsobarQuant and Mascot V2.4 (Matrix Science), Ethovision XT 14, AHCODA data analysis

For manuscripts utilizing custom algorithms or software that are central to the research but not yet described in published literature, software must be made available to editors and reviewers. We strongly encourage code deposition in a community repository (e.g. GitHub). See the Nature Portfolio guidelines for submitting code & software for further information.

Data

Policy information about availability of data

All manuscripts must include a data availability statement. This statement should provide the following information, where applicable:

- Accession codes, unique identifiers, or web links for publicly available datasets
- A description of any restrictions on data availability
- For clinical datasets or third party data, please ensure that the statement adheres to our policy

All data generated or analysed during this study are included in this published article (and its supplementary information files).

Field-specific reporting

Please select the one below that is the best fit for your research. If you are not sure, read the appropriate sections before making your selection.

- [x] Life sciences
- [ ] Behavioural & social sciences
- [ ] Ecological, evolutionary & environmental sciences

For a reference copy of the document with all sections, see nature.com/documents/nr-reporting-summary-flat.pdf

Life sciences study design

All studies must disclose on these points even when the disclosure is negative.

Sample size	For behavioral experiments samples size was determined by performing a power analysis, using our own results from N=5 animals.
Data exclusions	No data were excluded from the analyses
Replication	Data were reproduced by two independent scientists in the lab. For all analyses, scientists were blind to genotypes and treatments.
Randomization	Random allocation
Blinding	Investigators were blinded to the samples they analyzed.

Reporting for specific materials, systems and methods

We require information from authors about some types of materials, experimental systems and methods used in many studies. Here, indicate whether each material, system or method listed is relevant to your study. If you are not sure if a list item applies to your research, read the appropriate section before selecting a response.

Materials & experimental systems

n/a	Involved in the study	
[ ]	[x]	Antibodies
[x]	[ ]	Eukaryotic cell lines
[x]	[ ]	Palaeontology and archaeology
[ ]	[x]	Animals and other organisms
[x]	[ ]	Human research participants
[x]	[ ]	Clinical data
[x]	[ ]	Dual use research of concern

Methods

n/a	Involved in the study	
[x]	[ ]	ChIP-seq
[x]	[ ]	Flow cytometry
[x]	[ ]	MRI-based neuroimaging

Antibodies

Antibodies used

Atg16L1 E-10 Santa Cruz Sc-393274 1/1000
LC3B Santa Cruz Sc-376404 1/1000
LC3B Sigma L7543 1/1000
Atg13 Sigma SAB4200100 1/1000
MAP2 Synaptic systems 188004 1/100
FIP200 Cell signaling 12436 1/1000
Atg101 Abcam ab229235 1/1000
ULK1 Cell signaling 8054 1/1000
p62 Calbiochem DR1057 1/1000
WIPI2 Abcam Ab105459 1/1000
GluA2 (N-terminus) Alomone AGP-073 1/1000
PSD95 Invitrogen MA1-046 1/1000
Arc Synaptic Systems 156003 1/1000
SAR1A Invitrogen PA19124 1/1000
Atg13 pS318 Rockland 600-401-C49 1/1000
β III tubulin (Tuj1) Santa Cruz Sc-8005 1/5000
β III tubulin (Tuj1) Abcam Ab18207 1/1000
actin (2Q1055) Santa Cruz Sc58673 1/5000
Atg5 Abcam Ab109490 1/1000
Atg5 Novus NB110-53818 1/1000
GRP78Bip Abcam Ab21685 1/1000
cofilin-1 ProteinTech 66057-1-Ig 1/1000
GRIA2/3 Milipore AB1506 1/1000

CamKII alpha ThermoFischer 13-7300 1/1000
Dynamin-1 Abcam Ab52611 1/1000
Fyn (FYN-01) Invitrogen MA1-19331 1/1000
IL1RAPL1 Invitrogen PA5-96244 1/1000
IQSEC1 Invitrogen PA5-95835 1/1000
ITPKA Invitrogen PA5-85786 1/1000
KCC2 Invitrogen PA5-78544 1/1000
MYH10 Invitrogen PA5-88304 1/1000
PICK1 Invitrogen PA1-073 1/1000
SAP97 Invitrogen PA1-741 1/1000
alpha-Internexin ThermoFischer 32-3600 1/1000
Rab11B Novus biological NBP2-15085 1/1000
GluA2 Synaptic systems 182211 1/1000
PSD95 Invitrogen MA1-046 1/1000
Atg9α Novus Biologicals NBP2-67616 1/1000
EEA1 Abcam Ab2900 1/1000
Stx4 Synaptic systems SYSY110041 1/1000
Alix Cell Signalling 2171S 1/1000
TBP Abcam Ab61411 1/1000
LMAN1 Invitrogen PA1-074 1/1000
TGN46 Abcam Ab16059 1/1000
IgG Milipore 12-370 1/1000
Anti-rabbit Alexa 488; Abcam; Cat# ab150073
Anti-mouse Alexa 594; Abcam; Cat# ab150116
Anti-guinea pig Alexa 647; Abcam; Cat# ab150187
Anti-rabbit IgG (H+L); Jackson; Cat# 211-035-109; Lot: 143964
Anti-mouse IgG (H+L); Jackson; Cat# 715-035-150; Lot: 144459

Validation

Validation statement of each primary antibody of this study can be found in the manufacturer's website

Animals and other organisms

Policy information about studies involving animals; ARRIVE guidelines recommended for reporting animal research

Laboratory animals

Wildtype laboratory mice of a C57BL6 background were used. Atg5flox/flox and Thy1-CreERT2 mice were also on the same background.

Wild animals

N/A

Field-collected samples

N/A

Ethics oversight

FORTH Ethics Commitee, Greece and Affaires veterinaires Canton Vaud, Switzerland

Note that full information on the approval of the study protocol must also be provided in the manuscript.
