# Supplementary material for: Dendritic autophagy degrades postsynaptic proteins and is required for long-term synaptic depression in mice
Source: Nat Commun. 2022 Feb 3;13:680. doi: 10.1038/s41467-022-28301-z (PMC8814153; doi:10.1038/s41467-022-28301-z)
Supplement: Supplementary file 4 — Description of Additional Supplementary Files [file 41467_2022_28301_MOESM4_ESM.pdf]

Title: Supplementary data 1.
Description: Comparison of the autophagic vesicle (AV) content of hippocampal slices with that previously described in cell lines (Mancias *et al.*,).

Column A. List of all proteins identified in the AV-enriched fraction of hippocampal slices.
Column B. List of all proteins identified as AV content in cell lines (Mancias *et al.*,).
Column C. List of AV content proteins that are common between hippocampal slices and cell lines.
Column D. List of proteins that are uniquely identified in the hippocampal slice AV content.

Title: Supplementary data 2.
Description: Hippocampal slice autophagic vesicle (AV) content that shows differential abundance between control and LTD conditions.

Table includes all hippocampal slice AV-content proteins that are either increased or decreased in autophagic vesicles in the LTD condition, as compared to control. The classification in column L as hit, candidate or trend is based on the FDR values (<0.05, <0.2 and <0.6, respectively).
